# The impact of DNA damage response gene polymorphisms on therapeutic outcomes in late stage ovarian cancer

**DOI:** 10.1038/srep38142

**Published:** 2016-12-01

**Authors:** F. Guffanti, R. Fruscio, E. Rulli, G. Damia

**Affiliations:** 1 Department of Oncology, Laboratory of Molecular Pharmacology, IRCCS-Istituto di Ricerche Farmacologiche Mario Negri, Milan, Italy; 2 Clinc of Obstetrics and Gynecology, San Gerardo Hospital, Department of Medicine and Surgery, University of Milan-Bicocca, Italy; 3 Department of Oncology, Laboratory of Methodology for Clinical Research, IRCCS-Istituto di Ricerche Farmacologiche Mario Negri, Milan, Italy

## Abstract

Late stage epithelial ovarian cancer has a dismal prognosis. Identification of pharmacogenomic markers (i.e. polymorphisms) to stratify patients to optimize individual therapy is of paramount importance. We here report the retrospective analysis of polymorphisms in 5 genes (*ATM*, *ATR*, *Chk1*, *Chk2* and *CDK12)* involved in the cellular response to platinum in a cohort of 240 cancer patients with late stage ovarian cancer. The aim of the present study was to evaluate associations between the above mentioned SNPs and patients’ clinical outcomes: overall survival (OS) and progression free survival (PFS). None of the *ATM*, *ATR*, *Chk1* and *Chk2* polymorphisms was found to significantly affect OS nor PFS in this cohort of patients. Genotype G/G of *CDK12* polymorphism (rs1054488) predicted worse OS and PFS than the genotype A/A-A/G in univariate analysis. The predictive value was lost in the multivariate analysis. The positive correlation observed between this polymorphism and age, grade and residual tumor may explain why the *CDK12* variant was not confirmed as an independent prognostic factor in multivariate analysis.The importance of *CDK12* polymorphism as possible prognostic biomarker need to be confirmed in larger ovarian cancer cohorts, and possibly in other cancer population responsive to platinum agents.

Ovarian cancer is the fourth most common cancer and the leading cause of death in gynaecologic neoplasms^1^. The lack of symptoms and efficacious screening tests account for the fact that the majority of patients are diagnosed with an advanced tumor stage (stage III and IV). Despite the progress in surgical techniques and its remarkable tumor with cytotoxicity chemo-sensitivity, the rate of tumor recurrence is high, with <20% of the advanced stage patients surviving in long term^2^. Standard first line therapy consist of platinum-taxane therapy^3^. Platinum containing drugs are agents with different effects leading to cell death and among these, the DNA induced lesions have been correlated with citotoxicity^4^. DNA damage induces a cellular response which has the final aim to detect and remove the damage through the coordinated action of many different proteins involved in DNA repair, cell-cycle arrest, and apoptosis induction^5^. Inability to activate such processes has been variably associated to sensitivity/resistance to platinum containing drugs in different tumors, including ovarian cancer^6^,^7^.

The sequencing of the human genome together with the development and implementation of new high throughput technologies (so called “omics”) have greatly enhanced not only our knowledge on human cancer biology, but have opened up the ways to breakthroughs accelerating the road towards a personalized patient treatment. Changes at genetic level generally cause cellular phenotypic modifications that can partly explain the variability in response and toxicity to chemotherapy, including to regimens containing platinum^8^. A part from mutations causing pathological syndromes and possibly aberrant response to anticancer agents, there are many germinal variations in DNA sequence called polymorphisms^9^,^10^. These can be short tandem repeat, gene copy number variations or in more than 90% of the cases are single–nucleotide polymorphisms (SNPs). Unlike mutations, SNPs are very common, with a frequency of at least 1% in the population and generally they do not have deleterious clinical consequences, whatever type of SNP, i.e. synonymous (silent) or non- synonymous (amino-acid substitution). Nevertheless, they can be associated with changes in expression or function of the encoded protein, predisposing subjects to disease and/or possibly influencing their response to a given drug, both in terms of activity and toxicity^11^.

In this post genomic era, the possibility of individualizing cancer therapy by using molecular markers (SNPs) that predict the toxicity of chemotherapy has been pursued with some success^12^,^13^,^14^. More controversial are the results of SNPs in predicting the efficacy of anticancer agents^15^,^16^,^17^. We here report the retrospective analysis of 7 polymorphisms in 5 genes involved in the cellular response to cisplatin (DDP): *ATM*, *ATR*, *Chk1*, *Chk2* and *CDK12* in a cohort of 240 cancer patients with stage III/IV ovarian cancer underwent surgery and adjuvant chemotherapy. The aim of the present study was to evaluate associations between the above mentioned SNPs and patients’clinical outcome, overall survival (OS) and progression free survival (PFS).

## Results

### Patient’s characteristics

240 patients with stage III/IV ovarian carcinoma diagnosis were diagnosed from September 1979 to December 2004 at the Clinic of Obstetrics and Gynaecology, San Gerardo Hospital (Monza, Italy) and included in this study. The main characteristics of our patient population are summarized in Table 1. The mean age at diagnosis was 54.3 years; residual tumor size was >2 cm in 175 (72.9%) patients; the predominant hystotype was serous (77.9%) and poorly differentiated grade (64.6%). All patients were treated with platinum-based chemotherapy.

### Allele frequencies of studied polymorphisms

The *ATM*, *ATR*, *Chk1* and *Chk2* polymorphisms were selected based on their functional significance, on previous reports of association with cancer risk or clinical outcome or, in the case of *CDK12*, for the emerging role of this protein in influencing the response to therapy (Supplementary Table 1)^18^,^19^,^20^,^21^,^22^. As detailed in Table 2, all the polymorphisms were evaluable in most of the patient samples and the genotype frequencies of polymorphisms in *ATM* (rs664677 and rs664243), *ATR* (rs2229032 and rs2227928), *Chk1* (rs521102), *Chk2* (rs2267130), and *CDK1*2 (rs1054488) were equal to those predicted by the Entrez data base (hyyp:// www.ncbi.nlm.nhi.gov/sites/entrz?db=snp) for a Caucasian population.

### Progression free and overall survival analysis

At a median follow-up of 11.26 months, 186 patients had progression and 178 died. OS median was 3.88 months (IQR (inter quartile range) 2.23–9.49) and PFS median was 2.31 months (IQR 1.26–5.92). Age at diagnosis (HR = 1.03, 95% CI: 1.01–1.04;P < 0.001), residual tumor size (HR = 2.3, 95% CI: 1.67–3.16; P < 0.001), and grading (HR _(G2/3 vs G1/borderline)_ = 3.69, 95% CI: 1.73–7.87; p = 0.001) were the baseline covariates statistically correlated to OS; the same statistically significant correlations were detected considering PFS as endpoint: age at diagnosis (HR = 1.02, 95% CI: 1.01–1.02; P < 0.001), residual tumor size (HR = 2.11, 95% CI: 1.54–2.89; P < 0.001), grading (HR_(G2/3 vs G1and borderline)_ = 3.03, 95% CI: 1.55–5.92; p = 0.001) (Table 3).

*ATM* (rs664677 and rs664243), *ATR* (rs2229032 and rs2227928), *Chk1* (rs521102), and *Chk2* (rs2267130) genotypes did not affect either OS or PFS. The Kaplan-Meier curves for OS and PFS of the *ATM, ATR, Chk1,* and *Chk2* polymorphisms are plotted in Supplementary Figure 1, Supplementary Figure 2, and Supplementary Figure 3, respectively and Table 4 reports the Cox models results for the *ATM*, *ATR*, *Chk1,* and *Chk2* genetic variants.

**Figure 1:**
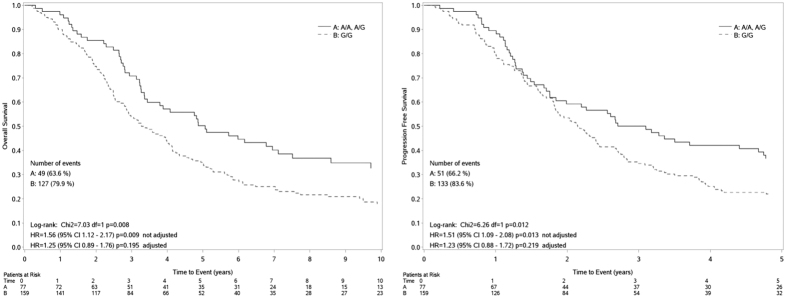
Overall Survival (left panel) and Progression Free Survival (right panel) curves by *CDK12* genotypes.

When the *CDK12* polymorphism was considered, the genotype G/G was found to be associated with a worse prognosis compared to the genotype A/A-A/G (HR_(G/G vsA/A, A/G)_ = 1.56, 95% CI: 1.12–2.17; P < 0.009; HR _(G/G vs A/A, A/G)_ = 1.51, 95% CI: 1.09–2.08, p = 0.013 for OS and PFS respectively) (Fig. 1). The median OS for those with genotype G/G was 3.3 months, compared to a median OS of 5.1months for those with A/A, A/G genotype (Fig. 1A). The median PFS for those with genotype G/G was 2.2 months, compared to a median PFS of 3.1 months for those with A/A, A/G genotype (Fig. 1B). The significance detected in the univariate analysis was lost in the multivariate analysis, after adjustment for grade, age and residual tumor. (HR _(G/G vs A/A, A/G)_ = 1.25, 95% CI: 0.89–1.76, p = 0.195;HR _(G/G vs A/A, A/G)_ = 1.23, 95% CI: 0.88–1.72, p = 0.013 for OS and PFS respectively); however the trend was again suggestive of an association between G/G and a worse prognosis.

### Correlation between the selected polymorphisms and patient characteristics

*CDK12* A/A, A/G genotypes statistically correlated with both low grade (p = 0.0028) and residual tumor <2 cm (p = 0.032) (Table 5). No correlations with other clinical variables were found. All the other SNPs did not show any correlation with the considered patient clinico-pathological characteristics (Supplementary Table 2).

### Effect of CDK12 polymorphism on CDK12 mRNA levels

For 70 of the 240 analyzed patients (29.1%) we were able to determine the expression of CDK12 mRNA in tumor. As shown in Supplementary Table 3, the CDK12 mRNA normalized expression level did not differ between the genotypes (G/G vs A/G and A/A; Kruscal-Wallis test, p = 0.554)

## Discussion

DNA damages are repaired by defined DNA repair pathways; there are however common sensor proteins that are activated by the presence of the damage and that coordinate the cellular response with the final aim to block cell cycle progression to allow repair, if the damage is reparable, and /or activate cell death, if the damage is too severe to be repaired^23^,^24^. The signalling network that coordinates the interaction and cross-talk among DNA repair, cell cycle and apoptosis is the DNA damage response (DDR) that represents an important barrier to oncogenesis^25^ and involves many proteins^5^,^26^,^27^,^28^,^29^. Among them, *ATR, ATM, Chk1* and *Chk2* play a crucial role^19^,^20^,^22^,^30^,^31^.

This study investigated the association of genomic variations in five different genes and outcomes in advanced stage ovarian cancer patients receiving chemotherapy. The selected genes code for proteins involved in key pathways of cellular response to different DNA damaging agents, including platinum^26^,^32^,^33^, that represents the gold standard in ovarian adjuvant therapy. Cisplatin (DDP) is an alkylating agent, whose cytotoxic effects are mainly related to its ability to cause DNA damage, that is repaired by the coordinated action of homologous recombination (HR), nucleotide excision repair (NER) and fanconi anemia (FA)^34^. The functional cellular DNA repair ability has been shown to modulate DDP sensitivity/resistance, being cells deficient in FA, HR and NER quite sensitive to the drug, while an over-activation of these pathways are associated with a lower drug activity^35^,^36^,^37^. As a general rule, gene polymorphisms do not have deleterious clinical consequences; however they can be associated with changes in expression or function of the encoded protein, predisposing subjects to disease and/or possibly influencing their response to a given drug, both in terms of activity and toxicity^11^. While polymorphisms of genes involved in specific DNA repair pathways have been studied in correlation with platinum-efficacy in many different patients cohort (i.e lung and ovarian patients)^18^,^37^,^38^,^39^,^40^, less information is available in upstream sensor DDR proteins. This is why we focused on the role of polymorphisms in genes involved in the initial activation of the cellular response to a given damage (*ATM/Chk2* and* ATR/Chk1* axis) and on *CDK12*, recently recognized as an important regulator of DDR in ovarian cancer^33^. None of the *ATM*, *ATR*, *Chk1* and *Chk2* polymorphisms was found to significantly affect OS nor PFS in ourcohort of advanced stage ovarian cancer patients treated with adjuvant chemotherapy, while genotype G/G of *CDK12* polymorphism (rs1054488) predicted worse OS and PFS than the genotype A/A-A/G in univariate analysis. Its predictive value was lost in multivariate analysis, even if the trend was again suggestive of an association between G/G and a worse prognosis.

No data on the association of these polymorphisms and response to therapy in ovarian cancer have been reported yet, while few data on the *ATM*, *ATR*, Chk1 and Chk2 SNPs under study have been reported in other tumor types. The *ATM* rs664143 polymorphism occurs at the G60A of intron 7 and has been postulated to be the binding site for intronic splicing enhancers and/or repressors leading to alter protein splicing^41^. The A allele in *ATM* rs664143 polymorphism has been associated with a lower DNA repair capacity^42^ and in a case-control study, it was found associated with increased risk of lung cancer compared to G allele^43^ and aggressiveness in prostate cancer^41^. No association with response to treatment in non-small cell lung cancer patients^44^ and with relapse free-survival in prostate cancer^41^ have been reported, in line with the data of the present manuscript. On the contrary,a significant reduced overall survival was associated with the heterozygous variant allele (HR = 2.35–95% CI 1.24–4.46; p = 0.007) in 119 patients with potentially resectable pancreatic cancer receiving neo-adjuvant therapy^21^. *ATR* is a gene encoding for a protein kinase important for the maintaining of genomic integrity^45^. Specifically, the kinase is activated by different damages that block the replication fork and activate Chk1 on Ser 345^46^. Both the selected *ATR* polymorphisms are missense variants causing a change in one single amino acid, preserving the protein length. These two genotype variants were investigated by Okazaki *et al*. in a cohort of pancreatic patients and only the rs2227928 C/C genotype was marginally associated with worse OS and lower success rate of tumor resection (p = 0.079 and 0.051, respectively)^21^, while no association was found with both OS and PFS in our patients population.

Chk1 and Chk2 are tyrosine kinases involved in the DDR pathway able to activate p53, modulate repair and block the cell cycle to favour damage repair^30^. Few data exist on the role of the *Chk1* and *Chk2* polymorphisms in response to therapy. Only, *Chk1*A/A genotype was found to be significant independent predictor of survival after adjusting for different clinical co-variates (sex, race, diabetes, CA19-9 and tumor resection; *P* = *0.007*) in pancreatic cancer patients^21^. We could not corroborate this finding in our cohort of late stage ovarian patients, possibly suggesting a different role of Chk1 in the response to platinum containing regimens in different tumor type (ovarian vs pancreatic tumor).

CDK12 is a cyclin dependent kinase involved in the regulation of transcription and in post-transcriptional mRNA processing through the phosphorylation of the C-terminal domain (CTD) of RNA polymerase II (RNAPII)^47^. CDK12 has been implicated in modulating the transcription of long and complex genes, i.e. genes involved in DNA repair^48^,^49^. It has been shown to be mutated in different tumortypes^33^ and the Cancer Genome Atlas Research Network (TGCA) revealed it to be mutated in 3% of the 316 high grade ovarian sequenced samples, with a prevalence similar to the one observedfor *BRCA-1* and *BRCA-2* (3.5 and 3.2% respectively)^50^. More importantly, these mutations have been shown to have functional consequences disabling the enzyme catalytic activity and hampering the ability of CDK12 to promote target gene expression^51^. In addition, cells expressing catalytic inactive CDK12 display functional defects in homologous recombination (HR), repair and depletion of CDK12 sensitized ovarian cells to cisplatin and PARP inhibitors^52^. Given this background, we wanted to explore the role, if any, of *CDK12* polymorphism in treatment response in ovarian cancer patients. The selected polymorphism is located in the 3′ UTRregion of *CDK1*2. As a general rule, the 3′ UTR sequences harbour regulatory motifs that influence mRNA turnover, stability, and localization governing in such a way the post-transcriptional gene regulation. To our knowledge no data have been published on the role *CDK12* polymorphisms in response to therapy in any tumor type. We found that the G/G genotype predicted a worse OS and PFS in univariate analysis than the A/A and A/G genotype. The positive correlation observed between this polymorphism and age, grade and residual tumor may explain why this *CDK12* variant was still present as a trend, but could not be confirmed as a significant independent prognostic factor in multivariate analysis, where the above mentioned characteristics maintained their predictive role. We tried to explore the functional consequence of the selected polymorphism on CDK12 mRNA expression level, as recently has been published on the predictive role of CDK12 mRNA level in OS of ovarian cancer patients^53^. However, we found that the different genotypes were not associated with a different CDK12 mRNA level, suggesting that other factors (i.e. protein translation, microRNAs, catalytic function) could be at the basis of the role of this *CDK12* polymorphism in being associated with OS.

The polymorphisms of genes involved in the upstreamof DDR investigated in this study were not associated with PFS and/or OS in this cohort of ovarian patients; in addition, our data highlight the importance of *CDK12* polymorphism as a possible prognostic biomarker in ovarian cancer. The relatively small sample size requires further studies to confirm these findings in ovarian patients; in addition, these data foster investigation of the role of this polymorphism in other cancer population responsive to platinum agents.

## Materials and Methods

### Patients and sample collection

Biopsies collected at primary surgery at the San Gerardo Hospital (Monza, Italy) were immediately frozen and stored at −80 °C. The collection and use of tumor samples were approved by the Ethics Committee of San Gerardo Hospital, and patients gave written informed consent and the study was carried out following the principles of the Declaration of Helsinki. Samples were comprised of >70% of tumor cells, as examined after hematoxylin and eosin staining.

### SNP selection and genotyping

DNA from tumor was extracted using the Maxwell 16 DNA Purification kit (Promega, Milan, Italy). DNA was amplified using Go Taq Hot Start Polymerase (Promega). The seven SNPs were studied using TaqMan SNP Genotyping assays (Applied Biosystems, Monza, Italy): *CDK12* (rs1054488), *ATM* (rs664677 and rs664143), *ATR* (rs2229032 and rs2227928), *Chk1* (rs521102), and *Chk2* (rs2267130) (Supplementary Table 1). These SNPs were selected based on the fact that these genes have been described to have a role in the cellular response to cisplatin^18^,^19^,^20^,^21^,^22^. PCR was performed in 384-well plates prepared with automatic liquid handling (epMotion 5075; Eppendorf, Milan, Italy).

### RNA isolation and Real Time PCR

Tumor fragments were homogenized in RNA lysis buffer in ice with an Ultra-turrax and RNA was purified using the Maxwell 16 LEV Simply RNA Kit (Promega). Retro-transcription to cDNA was done using the High Capacity cDNA Archive Kit (Applied Biosystem). Optimal primer pairs for *CDK12* (Forward 5′-tgtcacagataaacaagatgcac-3′, Reverse 5′-tgcaccaaaccagattctagc-3′) were chosen, spanning splice junctions, using PRIMER-3 software (http://frodo.wi.mit.edu/cgi-bin/primer3/primer3_www.cgi) and the specificity was verified by detecting single-band amplicon of the PCR product. Absolute copy numbers of mRNA were determined by real time RT-PCR (ABI-7900, Applied Biosystems) with the SYBR Green technique, using an EP Motion 5075 robot (Eppendorf). Standard curve was included for absolute quantification of mRNA. Data were normalized with the housekeeping cyclophilin gene (forward 5′- GACCCAACACAAATGGTTCC-3′, reverse 5′- TTTCACTTTGCCAAACACCA-3′).

### Statistical methods

A consecutive cohort of patients with ovarian cancer for which biological material was available was identified and retrospectively enrolled in this monocentric study. Baseline covariate distributions were summarized using descriptive statistics (median and range for continuous variables; absolute and percentage frequencies for categorical variables); t-test for continuous covariates and chi square test for categorical covariates were used to detect statistical association. Progression Free Survival (PFS) was defined as the time from the date of diagnosis up to the date of first progression or death from any cause, whichever came first. Subjects who have not progressed or died while on study were censored at the last disease assessment date. Overall survival (OS) was defined as the time from the date of diagnosis up to the date of death from any cause. Subjects who have not died while on study were censored at the last follow-up. Survival curves were estimated with the Kaplan-Meier method. Cox proportional hazards models were used for univariate and multivariate analysis to estimate and test demographic characteristics, clinical features, and biological parameters for their associations with PFS and OS. Results were expressed as Hazard Ratios (HRs) and their 95% confidence intervals (95% CIs). When the genotype frequency was lower than 20%, the polymorphism was analyzed combining the genotype with low number of patient with the heterozygous allele genotype. Statistical analyses were carried out using SAS version 9.2 (SAS Institute, Cary, NC).

## Additional Information

**How to cite this article**: Guffanti, F. *et al*. The impact of DNA damage response gene polymorphisms on therapeutic outcomes in late stage ovarian cancer. *Sci. Rep.* **6**, 38142; doi: 10.1038/srep38142 (2016).

**Publisher's note:** Springer Nature remains neutral with regard to jurisdictional claims in published maps and institutional affiliations.

## Supplementary Material

Supplementary Information

## Figures and Tables

**Table 1:** Clinical and histopathological characteristics of the patients under study.

	n	%
Age		
Median	54.3	
Q1-q3	46.5–64.9	
Stage		
III	214	89.17
IV	26	10.83
First line treatment		
Cisplatin	90	37.5
Cisplatin + Taxol	100	41.67
Taxol	2	0.83
Other	48	20
Grade		
Borderline	6	2.5
1	15	6.25
2	64	26.67
3	155	64.58
Histotype		
serous	187	77.92
endometrioid	22	9.17
clearcell	13	5.42
mucinous	8	3.33
undifferentiated	7	2.92
Other	3	1.25
Tumorresidual		
<2 cm	87	36.25
>2 cm	153	63.75
NED/micro	41	17.08
<1 cm	30	12.5
1-2 cm	16	6.67
2-5 cm	36	15
5-10 cm	52	21.67
>10 cm	65	27.08

**Table 2:** Expected and observed SNPs prevalence (%).

	Expected*	*Observed*	
	%	n	%
***ATM*** rs664677			
** **C/C	14,10	26	11,11
** **C /T	46,50	110	47,01
** **T/T	39,40	98	41,88
***ATM*** rs664143			
** **A/A	13,90	16	6,81
** **A/G	46,70	117	49,79
** **G/G	39,40	102	43,40
***ATR***rs2229032			
** **C/C	68,40	175	74,15
** **C /T	28,80	55	23,31
** **T/T	2,80	6	2,54
***ATR*** rs2227928			
** **A/A	16,90	33	13,87
** **A/G	48,90	110	46,22
** **G/G	34,20	95	39,92
***Chk1***rs521102			
** **A/A	25,40	75	32,33
** **A/G	49,10	91	39,22
** **G/G	25,40	66	28,45
***Chk2***rs2267130			
** **C/C	23,70	70	29,79
** **C /T	49,30	102	43,40
** **T/T	27,00	63	26,81
***CDK12***rs1054488			
** **A/A	2,40	7	2,97
** **A/G	27,80	70	29,66
** **G/G	69,80	159	67,37

^*^Ensembl Release 83 - December 2015.

**Table 3:** Prognostic evaluation of clinical and histopathological characteristics.

Variable	OS				PFS			
	Hazard Ratio	Lower 95% CI	Upper 95% CI	p value	Hazard Ratio	Lower 95% CI	Upper 95% CI	p value
Grade (2,3 vs borderline,1)	3.69	1.73	7.87	**0.001**	3.03	1.55	5.92	**0.001**
Tumor residual (>2 cm vs <2 cm)	2.41	1.72	3.38	**<0.001**	2.18	1.57	3.02	**<0.001**
Age (for 1 yearincrement)	1.03	1.01	1.04	**<0.001**	1.02	1.01	1.04	**<0.001**
Stage (IV vs III)	1.39	0.89	2.17	0.148	1.37	0.89	2.12	0.156
Hystotype (serousref.)	reference				reference			
endometrioid	1.03	0.6	1.76	0.911	1.02	0.62	1.69	0.94
other	1.33	0.86	2.05	0.203	1.21	0.79	1.86	0.373

**Table 4:** Univariate and multivariate Cox regression models by *ATM, ATR, Chk1* and *Chk2* genotypes.

Univariate Cox regression models								
	OS				PFS			
***ATM*****rs664677**								
(T/T vs C/T, C/C)	0.94	0.7	1.27	0.684	1	0.75	1.35	0.974
***ATM*****rs664143**								
(G/G vs A/G, A/A)	0.94	0.7	1.27	0.697	1	0.75	1.34	0.991
***ATR*****rs2229032**								
(C/C vs C/T, T/T)	0.92	0.66	1.29	0.634	0.83	0.6	1.15	0.266
***ATR*****rs2227928**								
(G/G vs A/G, A/A)	1.02	0.75	1.37	0.916	0.97	0.73	1.31	0.857
***Chk1*** **rs521102** (A/A)	reference				reference			
(A/G)	1.09	0.76	1.56	0.635	1.12	0.79	1.6	0.511
(G/G)	1.31	0.89	1.93	0.164	1.25	0.86	1.82	0.246
***Chk2*** **rs2267130** (C/C)	reference				reference			
(C/T)	1.05	0.74	1.48	0.804	0.95	0.68	1.34	0.786
(T/T)	0.9	0.6	1.33	0.585	0.8	0.54	1.17	0.251
**Multivariate Cox regression models**								
***ATM*****rs664677**								
(T/T vs C/T, C/C)	0.84	0.62	1.13	0.245	0.91	0.68	1.22	0.531
***ATM*****rs664143**								
(G/G vs A/G, A/A)	0.87	0.65	1.18	0.379	0.94	0.7	1.26	0.682
***ATR*****rs2229032**								
(C/C vs C/T, T/T)	1.03	0.74	1.45	0.852	0.91	0.66	1.27	0.591
*ATR* rs2227928								
(G/G vs A/G, A/A)	0.93	0.69	1.26	0.639	0.87	0.64	1.17	0.341
***Chk1*** **rs521102** (A/A)	reference				reference			
(A/G)	1.06	0.74	1.52	0.745	1.09	0.77	1.55	0.627
(G/G)	1.48	1	2.18	0.047	1.37	0.94	2	0.103
***Chk2*** **rs2267130** (C/C)	reference				reference			
(C/T)	1.08	0.76	1.54	0.656	0.99	0.7	1.4	0.946
(T/T)	1.01	0.68	1.5	0.971	0.87	0.59	1.28	0.482

HR: Hazard Ratio; CI: Confidential Interval.

**Table 5:** Associations between clinic-pathological patients characteristics and *CDK12* rs1054488 polymorphism.

Variable	*CDK12* rs1054488		Chi square test p value
	A/A, A/G (%)	G/G (%)	
**Grade**			
Borderline, 1	13 (16.88)	8 (5.03)	**0.0028**
2,3	64 (83.12)	151 (94.97)	
**Histology**			
Serous	57 (74.03)	127 (79.87)	
Endometrioid	9 (11.69)	13 (8.18)	0.5678
Other	11 (14.29)	19 (11.95)	
**Tumor residual**			
NED/micro	18 (23.38)	22 (13.84)	**0.0233**
<1 cm	13 (16.88)	16 (10.06)	
1-2 cm	1 (1.3)	15 (9.43)	
2-5 cm	13 (16.88)	23 (14.47)	
5-10 cm	18 (23.38)	33 (20.75)	
>10 cm	14 (18.18)	50 (31.45)	
**Age Mean years (std)**	52.61	55.42	t-test
	(14.36)	(12.65)	p = 0.1279

## References

[b1] Siegel R., Ma J., Zou Z. & Jemal A. Cancer statistics, 2014. CA Cancer J Clin 64, 9–29 (2014). 24399786 10.3322/caac.21208

[b2] Jayson G. C., Kohn E. C., Kitchener H. C. & Ledermann J. A. Ovarian cancer. Lancet 384, 1376–1388 (2014). 24767708 10.1016/S0140-6736(13)62146-7

[b3] Davis A., Tinker A. V. & Friedlander M. “Platinum resistant” ovarian cancer: what is it, who to treat and how to measure benefit? Gynecol Oncol 133, 624–631 (2014). 24607285 10.1016/j.ygyno.2014.02.038

[b4] Amable L. Cisplatin resistance and opportunities for precision medicine. Pharmacol Res 106, 27–36 (2016). 26804248 10.1016/j.phrs.2016.01.001

[b5] Roos W. P., Thomas A. D. & Kaina B. DNA damage and the balance between survival and death in cancer biology. Nat Rev Cancer 16, 20–33 (2016). 26678314 10.1038/nrc.2015.2

[b6] Haynes B., Saadat N., Myung B. & Shekhar M. P. Crosstalk between translesion synthesis, Fanconi anemia network, and homologous recombination repair pathways in interstrand DNA crosslink repair and development of chemoresistance. Mutat Res Rev Mutat Res 763, 258–266 (2015). 25795124 10.1016/j.mrrev.2014.11.005 PMC4369322

[b7] Martin L. P., Hamilton T. C. & Schilder R. J. Platinum resistance: the role of DNA repair pathways. Clin Cancer Res 14, 1291–1295 (2008). 18316546 10.1158/1078-0432.CCR-07-2238

[b8] Rodriguez-Vicente A. E. . Pharmacogenetics and pharmacogenomics as tools in cancer therapy. Drug Metabol Personal Ther 31, 25–34 (2016). 26863347 10.1515/dmpt-2015-0042

[b9] Brookes A. J. The essence of SNPs. Gene 234, 177–186 (1999). 10395891 10.1016/s0378-1119(99)00219-x

[b10] Stoneking M. Single nucleotide polymorphisms. From the evolutionary past. Nature 409, 821–822 (2001). 11236996 10.1038/35057279

[b11] Komar A. A. Silent SNPs: impact on gene function and phenotype. Pharmacogenomics 8, 1075–1080 (2007). 17716239 10.2217/14622416.8.8.1075

[b12] Liu X., Cheng D., Kuang Q., Liu G. & Xu W. Association of UGT1A1*28 polymorphisms with irinotecan-induced toxicities in colorectal cancer: a meta-analysis in Caucasians. Pharmacogenomics J 14, 120–129 (2014). 23529007 10.1038/tpj.2013.10 PMC3992871

[b13] Patel J. N. Cancer pharmacogenomics: implications on ethnic diversity and drug response. Pharmacogenet Genomics 25, 223–230 (2015). 25751395 10.1097/FPC.0000000000000134

[b14] Hertz D. L. & Rae J. Pharmacogenetics of cancer drugs. Annu Rev Med 66, 65–81 (2015). 25386932 10.1146/annurev-med-053013-053944

[b15] Rodriguez-Antona C. & Taron M. Pharmacogenomic biomarkers for personalized cancer treatment. J Intern Med 277, 201–217 (2015). 25338550 10.1111/joim.12321

[b16] Robert J., Le Morvan V., Giovannetti E. & Peters G. J. On the use of pharmacogenetics in cancer treatment and clinical trials. Eur J Cancer 50, 2532–2543 (2014). 25103456 10.1016/j.ejca.2014.07.013

[b17] Liu S. & Kurzrock R. Toxicity of targeted therapy: Implications for response and impact of genetic polymorphisms. Cancer Treat Rev 40, 883–891 (2014). 24867380 10.1016/j.ctrv.2014.05.003

[b18] Borchiellini D., Etienne-Grimaldi M. C., Thariat J. & Milano G. The impact of pharmacogenetics on radiation therapy outcome in cancer patients. A focus on DNA damage response genes. Cancer Treat Rev 38, 737–759 (2012). 22387145 10.1016/j.ctrv.2012.02.004

[b19] Guleria A. & Chandna S. ATM kinase: Much more than a DNA damage responsive protein. DNA Repair (Amst) 39, 1–20 (2016). 26777338 10.1016/j.dnarep.2015.12.009

[b20] O’Connor M. J. Targeting the DNA Damage Response in Cancer. Mol Cell 60, 547–560 (2015). 26590714 10.1016/j.molcel.2015.10.040

[b21] Okazaki T. . Single-nucleotide polymorphisms of DNA damage response genes are associated with overall survival in patients with pancreatic cancer. Clin Cancer Res 14, 2042–2048 (2008). 18381943 10.1158/1078-0432.CCR-07-1520 PMC2423806

[b22] Weber A. M. & Ryan A. J. ATM and ATR as therapeutic targets in cancer. Pharmacol Ther 149, 124–138 (2015). 25512053 10.1016/j.pharmthera.2014.12.001

[b23] Damia G. & Broggini M. Cell cycle checkpoint proteins and cellular response to treatment by anticancer agents. Cell Cycle 3, 46–50 (2004). 14657665

[b24] Damia G. & D’Incalci M. Targeting DNA repair as a promising approach in cancer therapy. Eur J Cancer 43, 1791–1801 (2007). 17588740 10.1016/j.ejca.2007.05.003

[b25] Bartkova J. . DNA damage response as a candidate anti-cancer barrier in early human tumorigenesis. Nature 434, 864–870 (2005). 15829956 10.1038/nature03482

[b26] Jackson S. P. & Bartek J. The DNA-damage response in human biology and disease. Nature 461, 1071–1078 (2009). 19847258 10.1038/nature08467 PMC2906700

[b27] Kachalaki S., Ebrahimi M., Mohamed Khosroshahi L., Mohammadinejad S. & Baradaran B. Cancer chemoresistance; biochemical and molecular aspects: a brief overview. Eur J Pharm Sci 89, 20–30 (2016). 27094906 10.1016/j.ejps.2016.03.025

[b28] Lopez-Martinez D., Liang C. C. & Cohn M. A. Cellular response to DNA interstrand crosslinks: the Fanconi anemia pathway. Cell Mol Life Sci (2016). 10.1007/s00018-016-2218-x PMC4951507 27094386

[b29] Tian H. . DNA damage response–a double-edged sword in cancer prevention and cancer therapy. Cancer Lett 358, 8–16 (2015). 25528631 10.1016/j.canlet.2014.12.038

[b30] Carrassa L. & Damia G. Unleashing Chk1 in cancer therapy. Cell Cycle 10, 2121–2128 (2011). 21610326 10.4161/cc.10.13.16398

[b31] Manic G., Obrist F., Sistigu A. & Vitale I. Trial Watch: Targeting ATM-Chk2 and ATR-Chk1 pathways for anticancer therapy. Mol Cell Oncol 2, e1012976 (2015). 27308506 10.1080/23723556.2015.1012976 PMC4905354

[b32] Woods D. & Turchi J. J. Chemotherapy induced DNA damage response: convergence of drugs and pathways. Cancer Biol Ther 14, 379–389 (2013). 23380594 10.4161/cbt.23761 PMC3672181

[b33] Chila R., Guffanti F. & Damia G. Role and therapeutic potential of CDK12 in human cancers. Cancer Treat Rev 50, 83–88 (2016). 27662623 10.1016/j.ctrv.2016.09.003

[b34] Siddik Z. H. Cisplatin: mode of cytotoxic action and molecular basis of resistance. Oncogene 22, 7265–7279 (2003). 14576837 10.1038/sj.onc.1206933

[b35] Damia G., Imperatori L., Stefanini M. & D’Incalci M. Sensitivity of CHO mutant cell lines with specific defects in nucleotide excision repair to different anti-cancer agents. Int J Cancer 66, 779–783 (1996). 8647649 10.1002/(SICI)1097-0215(19960611)66:6<779::AID-IJC12>3.0.CO;2-Z

[b36] Tavecchio M. . Role of homologous recombination in trabectedin-induced DNA damage. Eur J Cancer 44, 609–618 (2008). 18243687 10.1016/j.ejca.2008.01.003

[b37] Macerelli M. . Can the response to a platinum-based therapy be predicted by the DNA repair status in non-small cell lung cancer? Cancer Treat Rev 48, 8–19 (2016). 27262017 10.1016/j.ctrv.2016.05.004

[b38] Garcia-Campelo R., Alonso-Curbera G., Anton Aparicio L. M. & Rosell R. Pharmacogenomics in lung cancer: an analysis of DNA repair gene expression in patients treated with platinum-based chemotherapy. Expert Opin Pharmacother 6, 2015–2026 (2005). 16197356 10.1517/14656566.6.12.2015

[b39] Macerelli M. . Can the response to a platinum-based therapy be predicted by the DNA repair status in non-small cell lung cancer? Cancer Treat Revin press (2014). 10.1016/j.ctrv.2016.05.004 27262017

[b40] Caiola E. . DNA-damage response gene polymorphisms and therapeutic outcomes in ovarian cancer. Pharmacogenomics J 13, 159–172 (2013). 22158331 10.1038/tpj.2011.50

[b41] Browning R. E. t. . ATM polymorphism IVS62+60G>A is not associated with disease aggressiveness in prostate cancer. Urology 67, 1320–1323 (2006). 16765197 10.1016/j.urology.2005.12.012

[b42] Shin A. . Genotype-phenotype relationship between DNA repair gene genetic polymorphisms and DNA repair capacity. Asian Pac J Cancer Prev 9, 501–505 (2008). 18990028

[b43] Kim J. H. . Genetic polymorphisms of ataxia telangiectasia mutated affect lung cancer risk. Hum Mol Genet 15, 1181–1186 (2006). 16497724 10.1093/hmg/ddl033

[b44] Su D. . Genetic polymorphisms and treatment response in advanced non-small cell lung cancer. Lung Cancer 56, 281–288 (2007). 17222938 10.1016/j.lungcan.2006.12.002

[b45] Cimprich K. A. & Cortez D. ATR: an essential regulator of genome integrity. Nat Rev Mol Cell Biol 9, 616–627 (2008). 18594563 10.1038/nrm2450 PMC2663384

[b46] Awasthi P., Foiani M. & Kumar A. ATM and ATR signaling at a glance. J Cell Sci 128, 4255–4262 (2015). 26567218 10.1242/jcs.169730

[b47] Jeronimo C., Collin P. & Robert F. The RNA Polymerase II CTD: The Increasing Complexity of a Low-Complexity Protein Domain. J Mol Biol 428, 2607–2622 (2016). 26876604 10.1016/j.jmb.2016.02.006

[b48] Blazek D. . The Cyclin K/Cdk12 complex maintains genomic stability via regulation of expression of DNA damage response genes. Genes Dev 25, 2158–2172 (2011). 22012619 10.1101/gad.16962311 PMC3205586

[b49] Kohoutek J. & Blazek D. Cyclin K goes with Cdk12 and Cdk13. Cell Div 7, 12 (2012). 22512864 10.1186/1747-1028-7-12 PMC3348076

[b50] Integrated genomic analyses of ovarian carcinoma. Nature 474, 609–615 (2011). 21720365 10.1038/nature10166 PMC3163504

[b51] Ekumi K. M. . Ovarian carcinoma CDK12 mutations misregulate expression of DNA repair genes via deficient formation and function of the Cdk12/CycK complex. Nucleic Acids Res 43, 2575–2589 (2015). 25712099 10.1093/nar/gkv101 PMC4357706

[b52] Joshi P. M., Sutor S. L., Huntoon C. J. & Karnitz L. M. Ovarian cancer-associated mutations disable catalytic activity of CDK12, a kinase that promotes homologous recombination repair and resistance to cisplatin and poly(ADP-ribose) polymerase inhibitors. J Biol Chem 289, 9247–9253 (2014). 24554720 10.1074/jbc.M114.551143 PMC3979363

[b53] Bajrami I. . Genome-wide profiling of genetic synthetic lethality identifies CDK12 as a novel determinant of PARP1/2 inhibitor sensitivity. Cancer Res 74, 287–297 (2014). 24240700 10.1158/0008-5472.CAN-13-2541 PMC4886090

