# Supplementary material for: The impact of DNA damage response gene polymorphisms on therapeutic outcomes in late stage ovarian cancer
Source: Sci Rep. 2016 Dec 1;6:38142. doi: 10.1038/srep38142 (PMC5131275; doi:10.1038/srep38142)
Supplement: Supplementary Information [file srep38142-s1.pdf]

The impact of DNA damage response gene polymorphisms on therapeutic outcomes in late stage ovarian cancer.

Guffanti F[1], Fruscio R[2], Rulli E[3*] and Damia G[1*].

[1]Department of Oncology, Laboratory of Molecular Pharmacology, IRCCS-Istituto di RicercheFarmacologiche Mario Negri, Milan, Italy.[2]Clinc of Obstetrics and Gynecology, San Gerardo Hospital, Department of Medicine and Surgery, University of Milan-Bicocca, Italy. [3]Department of Oncology, Laboratory of Methodology for Clinical Research, IRCCS-Istituto di RicercheFarmacologiche Mario Negri, Milan, Italy.

Legends to Supplementary Figures

Supplementary Figure 1. Overall survival and Progression Free Survival curves by ATM genotypes.

Supplementary Figure 2. Overall survival and Progression Free Survival curves byATR genotypes.

Supplementary Figure 3. Overall survival and Progression Free Survival curves by CHK1 (upper panels) and CHK2 (lower panels) genotypes.

Supplementary Table 1. SNPs evaluated.

Gene	*Chromosome*	*Most severe consequence*	*Ref. SNP ID n°*	*MFA*
ATM	11	T to C transition: intron variant	rs664677	0,35 (C)
	11	G to A transition: 3 prime UTR variant	rs664143	0,37 (A)
ATR	3	C to T transition: missense variant	rs2229032	0,10 (T)
	3	G to A transition: missense variant	rs2227928	0,40 (A)
CHEK1	11	G to A transition: intron variant	rs521102	0,44 (A)
CHEK2	22	T to C transition: intron variant	rs2267130	0,31 (C)
CDK12	17	G to A transition: 3 prime UTR variant	rs1054488	0,22 (A)

MFA= minor allele frequency

ATM rs664677

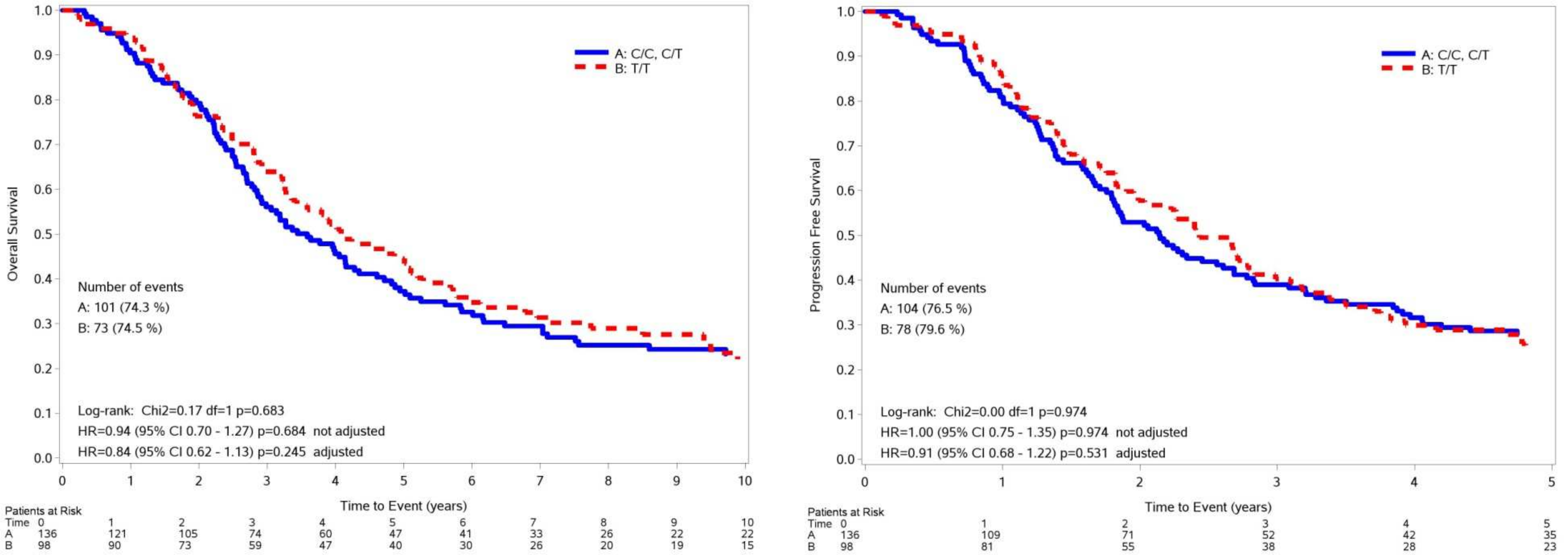

ATM rs664143

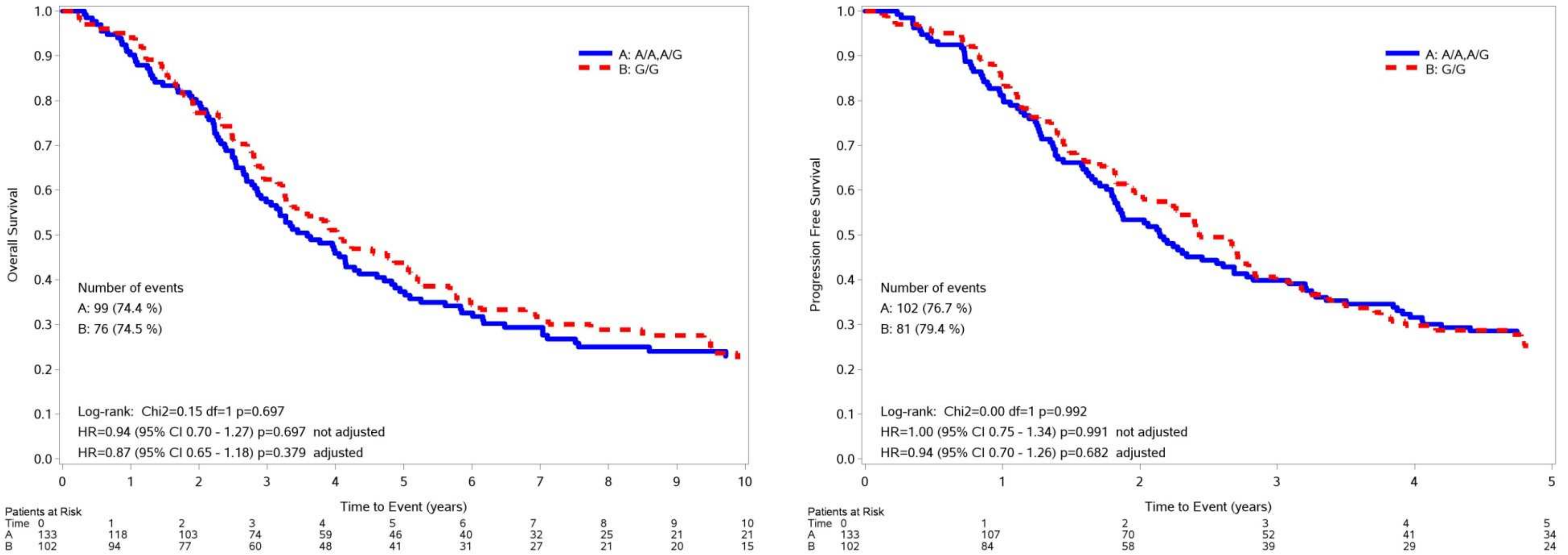

Supplementary Figure 1

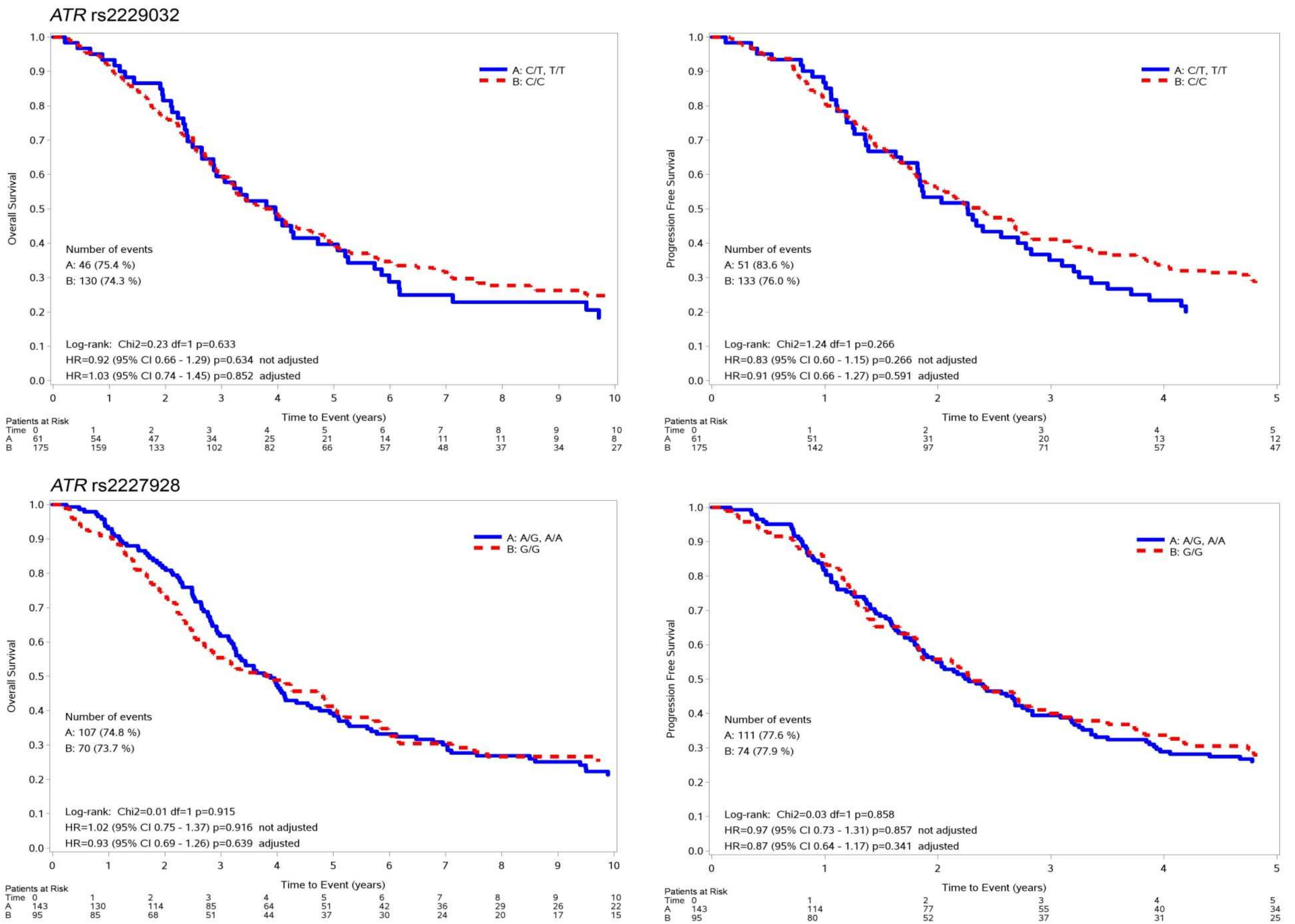

Supplementary Figure 2

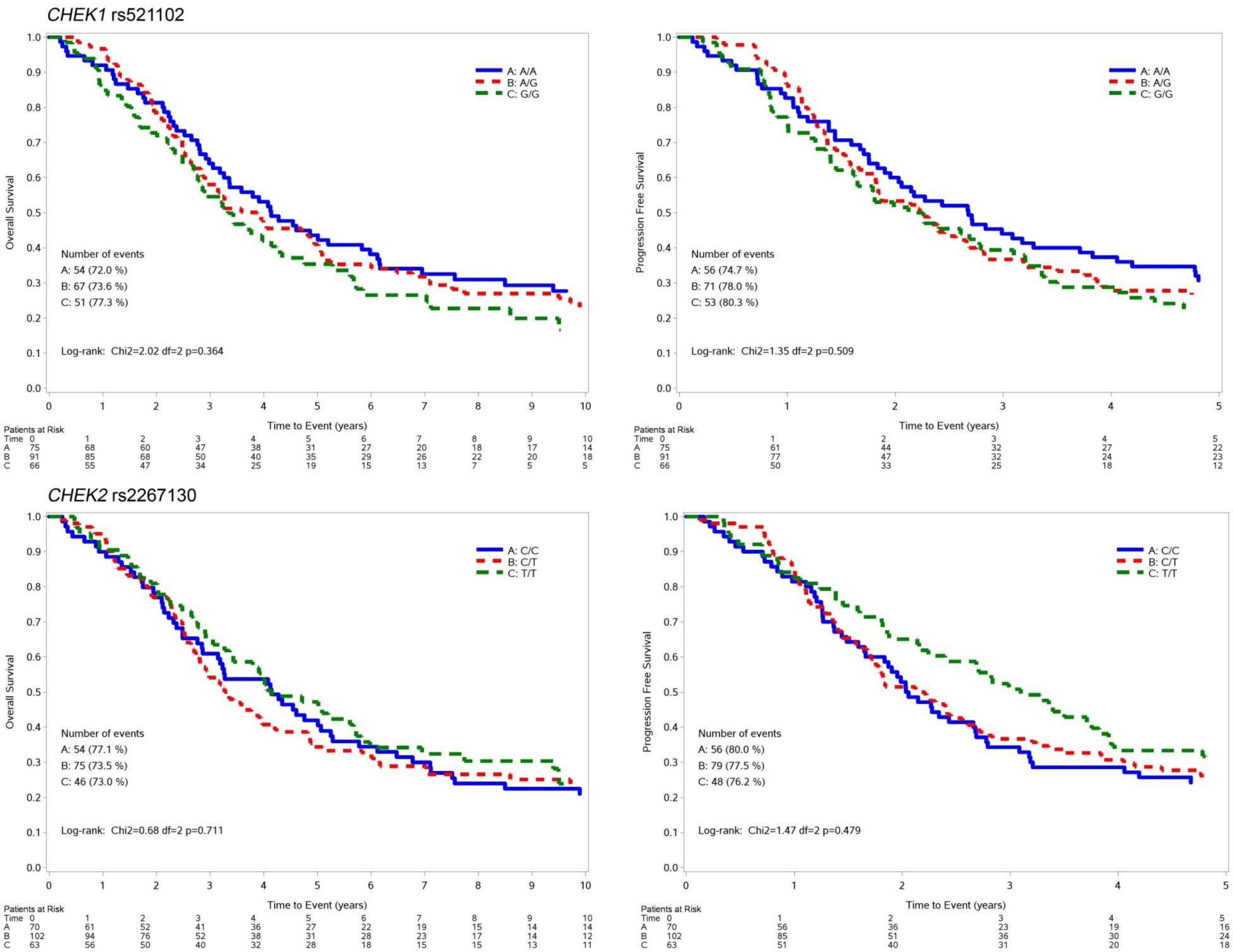

Supplementary Figure 3
